# Graphene Oxide (GO): A Promising Nanomaterial against Infectious Diseases Caused by Multidrug-Resistant Bacteria

**DOI:** 10.3390/ijms23169096

**Published:** 2022-08-13

**Authors:** Ida M. J. Ng, Suhaili Shamsi

**Affiliations:** Laboratory of Animal Biochemistry and Biotechnology, Department of Biochemistry, Faculty of Biotechnology and Biomolecular Sciences, Universiti Putra Malaysia, Serdang 43400, Malaysia

**Keywords:** graphene oxide, infectious disease, antibacterial, multidrug-resistant, mechanism of action

## Abstract

Infectious diseases are major threat due to it being the main cause of enormous morbidity and mortality in the world. Multidrug-resistant (MDR) bacteria put an additional burden of infection leading to inferior treatment by the antibiotics of the latest generations. The emergence and spread of MDR bacteria (so-called “superbugs”), due to mutations in the bacteria and overuse of antibiotics, should be considered a serious concern. Recently, the rapid advancement of nanoscience and nanotechnology has produced several antimicrobial nanoparticles. It has been suggested that nanoparticles rely on very different mechanisms of antibacterial activity when compared to antibiotics. Graphene-based nanomaterials are fast emerging as “two-dimensional wonder materials” due to their unique structure and excellent mechanical, optical and electrical properties and have been exploited in electronics and other fields. Emerging trends show that their exceptional properties can be exploited for biomedical applications, especially in drug delivery and tissue engineering. Moreover, graphene derivatives were found to have in vitro antibacterial properties. In the recent years, there have been many studies demonstrating the antibacterial effects of GO on various types of bacteria. In this review article, we will be focusing on the aforementioned studies, focusing on the mechanisms, difference between the studies, limitations and future directions.

## 1. Introduction

**Figure 1:**
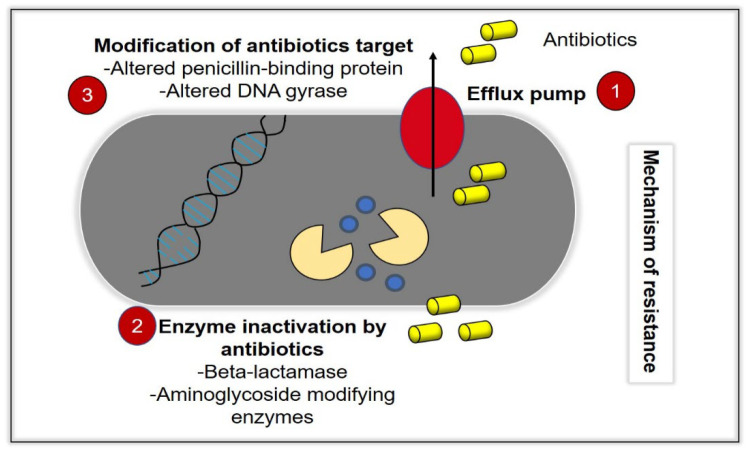
Bacteria acquire resistance toward antibacterial agents (antibiotics) through various mechanisms. (1) Efflux pump activity, in which the intracellular antibiotics are removed from the bacteria cells, which prevents the accumulation of antibiotics at its therapeutic concentrations, intracellularly. (2) Inactivation of enzymes, such as beta-lactamase, and (3) modification of antibiotics targets, such as alterations of the penicillin-binding protein (ABP) and DNA gyrase.

Infectious diseases represent a continuous and major threat to the worldwide suspension, and recently, they have become the main cause of enormous morbidity and mortality in all parts of the world [1]. The number of infectious diseases that caused by the growing of drug-resistant bacteria is ascending time by time, and the increased number of hospitalized patients with immunodeficiency has resulted in an increase of severe and invasive infections [2]. Due to these problems, many antibacterial agents exist for use against a wide range of infectious diseases, which can either be synthetic, plant and animal in origin or chemically modified natural compounds [3]. However, these bacteria acknowledge a remarkable effectiveness to adapt, survive and evolve by developing resistance against antibacterial compounds, which leads to the global spread of resistant microorganisms towards antibiotics [4]. While antibiotics supposedly become effective means to control such infections, antibiotic overuse triggers the spread of resistant strains in the population. As a result, many strains of dangerous bacterial pathogens nowadays are resistant to antibiotics, with some strains combining even multiple resistances to different antibiotics [5]. In the current era of multidrug resistance, in which bacteria are gaining resistance to many antimicrobial agents, it is becoming very difficult for healthcare workers to treat patients, leading to severe morbidity and mortality [6]. With the current increase in prevalence of MDR bacteria, there will be no more efficient antibiotics by 2050 [7]. The process of drug discovery and clinical trials of new antibacterial drugs take a long time; hence, only a few new agents have recently been approved and are available for use [8]. The emergence and spread of bacteria resistant to multiple antibacterial agents (so-called “superbugs”), due to mutations in the pathogens and overuse of antimicrobials, should be considered as a cause that triggers serious concern [6]. Commonly, the bacteria can acquire extrachromosomal genetic elements that contain the gene encoding the virulence factor leading to antimicrobial resistance. This occurs via horizontal gene transfer. Some defence mechanisms (Figure 1) include the difference in bacterial cell wall compositions, production of degrading enzymes against antibiotics, action of efflux pumps and modification of antibiotic targets leading to tolerance and resistance of the therapy [7]. In this review, we will discuss GO as a potential development for antibacterial therapeutics. This review includes the antibacterial mechanism of GO, the physicochemical properties of GO and its biocompatibility in vitro and in vivo, as well as the limitations and future directions.

## 2. Graphene Oxide

A plethora of studies have demonstrated the potential future application of nanomaterials in various disciplines. Nanomaterials can be employed in modality treatments, including targeted drug delivery systems, as well as integration into nanoscale probes for bioimaging, attributed to its unique physiochemical properties, enhanced durability and versatility. Recent advancements in technology coupled with researchers exploring nanomaterial applications in the health industry, have led to new innovative and promising solutions to problems that conventional tools have yet to solve. However, nanomaterial research has also raised new concerns, especially regarding its biocompatibility, which have prompted studies to assess the potential toxicity of nanomaterials.

Graphene oxide (GO) has been chosen as the nanomaterial of interest in the present review due to its tremendous potential. Graphene oxide (GO), an oxidized form of graphene, is regarded to be more superior to graphene with regards to application in drug delivery due to the presence of functional groups that could allow the binding of different compounds, especially water-insoluble drugs. The highly oxidized structure of GO could also provide better biocompatibility and permeability in the aqueous environment common within the human body, allowing it to be more effective in the targeted drug delivery system [9]. GO possessed the highest antimicrobial activity rather than another graphene-derived material [10]. In recent years, there have been many studies demonstrating the antibacterial effects of GO on various types of bacteria, including Gram-positive and Gram-negative bacteria, as shown in the table below. The antibacterial activities shown in these studies employed different experimental methods and data presentation, such as the zone of inhibition, colony counting and methods to indicate and quantify viable and dead cells by means of metabolic activity and fluorescent DNA. These studies also used different concentrations of GO and time of incubation, which is not possible to compare between the studies. In addition, these studies also could not conclude if the antibacterial activity is more efficient in Gram-positive or -negative bacteria. Based on the studies, it may be concluded that the treatment conditions depend on various factors, including amount, type, size and virulence of the bacteria. It can be seen that the antibacterial activity of GO is well-established, as many studies have reported its time and concentration-dependent effects (Table 1). However, it is still unknown and inconclusive whether GO is more effective towards Gram-positive or Gram-negative bacteria. It is imperative that we fully understand the antibacterial effects of GO in established biological models for further development of GO in clinical settings.

Furthermore, there has also been a handful of studies demonstrated that the effect of GO is effective both in Gram-positive and Gram-negative, according to their results. It was found that a study claimed that the thin peptidoglycan layer of the Gram-negative *E. coli* is easily penetrated by GO [15]. Interestingly, SEM in another showed the penetration of the cell membrane of *S. aureus* and claimed the antibacterial activity was more potent in Gram-positive bacteria, which has a thick peptidoglycan layer. This study also mentioned that *E. coli* has porins, which block the entrance of GO [13]. Another study also mentioned that the resistance of the Gram-negative *E. coli* is attributed to the presence of an outer membrane layer [25]. On the other hand, another study mentioned that the thickness of peptidoglycan activity does not affect the antibacterial activity of GO [26]. Various studies have shown the antibacterial activity of GO on different types of bacteria. Hence, these results are inconclusive for making a general rule on which type of bacteria is more potent in GO [27]. This has also been mentioned in a previous review discussion [28].

## 3. Mechanisms of Action

**Figure 2:**
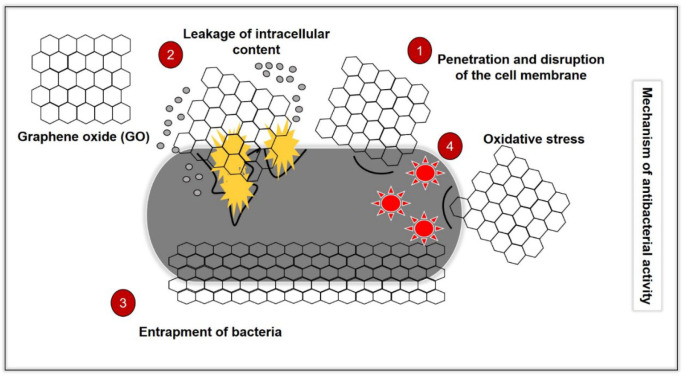
Illustration of the antibacterial mechanisms of GO, which consist of (1) penetration and disruption of the bacterial cell membrane, (2) leakage of the intracellular content following penetration and disruption of the cell membrane, (3) oxidative stress by the generation of reactive oxygen species (ROS0 and depletion of the antioxidant in the bacteria) and (4) bacteria entrapping (wrapping effect) by GO.

It well-known that GO demonstrates a different antibacterial mechanism than conventional antibiotics (Figure 2). The mechanisms exhibited by common MDR bacteria against antibiotics have been discussed in another review [7]. Hence, in this section, we will discuss the antibacterial mechanisms of GO and the limitations between the studies. This knowledge can forge the pathway for future studies on the development of GO as an antimicrobial agent.

### 3.1. Disruption of Bacteria Cell Membrane

A study has demonstrated the antibacterial activity of GO nanowalls against *S. aureus* and *E. coli*, which was attributed to the direct contact of the very sharp edges (nano-knives) on the nanowalls and the bacteria. In this study, SEM showed that the nanowalls that were almost perpendicular to the substrate that it was deposited on provided extremely sharp edges, allowing efficient interaction with the bacteria. The leakage of intracellular components also confirmed the cell membrane damage by the sharp edges of the GO nanowalls, which was more effective on *S. aureus* than *E. coli*. It was mentioned to be due to the lack of an outer membrane on *S. aureus* [25]. Interestingly, another study also showed that controlled vertically aligned GO nanosheets penetrated and disrupted the membrane of *E. coli*. In this study, GO nanoflakes suspension was used [29]. Thus, it is suggested that it could be due to the availability of more sharp edges in the suspension than that of being coated as GO is more freely mobile. This was supported by two other studies, which demonstrated that GO vertically oriented to the cell membrane is more ready for penetration due to it being able to overcome the energy barrier [30,31]. Moreover, studies with simulations have also reported that the GO nanosheets would enter a fluctuation or swinging mode so that they will find a diving force to overcome the energy barrier before piercing through the cell membrane [32,33]. In addition, another study also mentioned that vertical GO from approximately 3.5–4.7 nm of the bacteria membrane surface would always spontaneously penetrate through the cell membrane. Furthermore, after penetrating into the membrane, simulation experiments described the mechanism of GO disrupting the membrane by phospholipid extraction. This was said to be due to the hydrophobic interactions of the phospholipids and the hydrophobic “sp^2^” regions on the GO. It was also claimed that a larger GO (500 nm) demonstrated stronger antibacterial effects due to stronger cutting effects. Moreover, a larger GO also gives a larger hydrophobic region, which enhances the phospholipid extraction activity [33]. Another study also mentioned the importance of “sp^2^” regions in interactions with the phospholipid membrane [24]. Interestingly, SEM images from one study showed that a smaller GO (1295 nm) exhibits enhanced cutting effects on *S. mutans*. This is suggested to be attributed to a smaller area with more sharp edges exposed [16]. Nevertheless, 500 nm [33] is smaller than 1295 nm [16]. Another study also reported that smaller GO nanosheets enable the blade effect of sharp edges as its predominant antibacterial mechanism [34]. As mentioned above, these claims are due to researchers synthesising GO in different range of sizes. A study demonstrated that the presence of electrolytes in the GO suspension would prevent the activity of sharp edges due to reacting with the ions, forming aggregates [35]. It was said that the aggregation of GO increases the surfaces energy, thereby preventing the cutting action of the sharp edges. This study also demonstrated that the optimum concentration for the action of sharp edges is below 6 µg/mL when GO is suspended in any solution. However, as the concentration increases, antibacterial activity is only observed in GO dispersed in water. This was further confirmed by the observation of cellular debris and change in the morphology of bacteria on the AFM [34]. In addition to this, quantum mechanics calculations also indicated that further oxidation on the previous oxidised sites could significantly decrease the energy barrier [33]. In addition, another simulation study also reported that the degree of oxidation of GO affects its position within the phospholipid bilayer [31]. One other study mentioned than larger GO nanosheets with a higher degree of oxidation could cause larger and more irregular membrane perturbation [32]. In addition, another study also suggested that electron transfer from the membrane of *E. coli* to the oxygen functional groups of GO coated on metals leads to a loss of membrane integrity [36]. Moreover, another study also reported the availability of peaks or sharp edges promotes a charge transfer, thereby leading to destruction of the cell membrane. This was made evident by the increase in uptake of PI, a membrane impermeable dye, by the bacteria [18]. Therefore, it is important to consider these factors in the design and synthesis of GO.

In another study, SEM observation revealed the penetration of GO into *E. coli* with 50 µg/mL of treatment, while 100 µg/mL GO caused intracellular leakage. GO also increased the membrane peroxidase activity, which enhances penetration into *E. coli* in a time and concentration-dependent manner [15]. One other study also showed that 50–200 µg/mL of GO caused the membrane perturbation of *P. syringae* and *X. campestris pv. Undulosa* [37]. Furthermore, another study has reported the disruption of the membrane of *E. coli* caused by smaller GO-coated nanosheets (0.01 µm^2^) at 200 µg/mL. This was indicated by the increase in fluorescent propidium iodide. This was further confirmed by the change in bacteria morphology, as observed by the microscopy analysis [38]. Studies have also mentioned that the difference in efficiency of GO on the Gram-positive and -negative bacteria could be attributed to the difference in the cell membrane structure having roles in protecting the bacteria from the sharp edges of GO [39,40]. Another study also mentioned that piercing and lacerations of the phospholipid bilayer is more effective on Gram-negative bacteria, attributed to the thin peptidoglycan layer [18].

Based on previous studies, the antibacterial mechanisms are not generalised to a specific bacteria type or classification [27]. This could be due to the different laboratorial experimental conditions used. It is also widely accepted in various studies that the GO nanosheets undergo a series of mechanisms mentioned above in order to exhibit potent antibacterial activity. This can be seen by the overlapping studies mentioned reporting different antibacterial mechanisms. Initially, the GO nanosheets must be able to make direct contact with the bacteria. For this interaction to occur, the physicochemical properties are extremely crucial, for example, the alignment, functional group exposed, dispersibility and size. Studies have shown how these factors are able to affect the interaction of bacteria and GO-based materials [41]. Then, the GO can either cut and/or wrap the bacteria, depending on the physicochemical properties that will be reviewed below. Finally, GO will induce oxidative stress-inhibiting cellular functions and components, leading to cell death. For example, a study has shown that the functional groups and electronic properties of the GO nanosheets contribute to the its wrapping ability on *E. coli* and oxidative potential [42]. Nonetheless, oxidative stress can still be induced without physical contact. In general, most studies report a combination of mechanisms giving potent antibacterial activity. Moreover, there are also a few studies claiming that GO is able to inhibit bacterial biofilm formation [14,43]. Another study has also demonstrated the biofilm inhibitory effect of GO on *S. aureus* at 10 µg/mL and 100 µg/mL [44]. Several studies have also claimed the suicidal activity as an antibacterial mechanism. This has been discussed in a previous review [45]. However, more studies would need to be done on these mechanisms [28].

### 3.2. Bacteria Entrapping (Wrapping) Effect

A recent study has reported that larger-sized GO have a stronger entrapping ability [16]. In this study, SEM revealed GO with larger sizes ranging from 2015 to 4544 nm exhibited an entrapping effect on *S. mutans*. Furthermore, an SEM observation in a previous study has shown the entrapping effect of GO (0.31 ± 0.2 µm) on *E. coli* [41]. In addition to this, it was also reported in another study that larger-sized GO (0.65 µm^2^) possessed a stronger entrapping effect on *E. coli*. However, this effect was only bacteriostatic [38]. These findings were also observed in previous studies. After sonication to release the bacteria from the nanoparticles, the bacteria were able to proliferate once again [46,47,48]. Interestingly, another study showed the entrapping ability of GO nanoflakes (450–870 nm) on Mycobacterium smegmatis to be bacteriostatic [49]. One other study also mentioned that larger GO (>0.4 µm^2^) exhibited stronger antibacterial effects on *E. coli*, as they have a better bacterial entrapping ability, which is observed by AFM. This study described the inhibition of nutrient uptake due to bacteria being trapped within the GO, leading to cell death [24]. A handful of studies have claimed that larger GO exhibited a stronger entrapping effect. However, the effects of the GO sizes on this mechanism are not conclusive. This could be due to several reasons, as suggested below:

1. The method of size measurement used is different (area and diameter).
2. The difference in ranges of the GO sizes. For example, one study may synthesise nanosize GO, while, in another study, microsized GO is used.
3. The different types of bacteria with different sizes and concentrations used.

One study has described the role of GO nanosheets with peaks and terrains in the bacteria-entrapping activity. It was mentioned that the entrapping effect was enhanced when the size of the bacteria matches the deep terrains of GO, which led to the degradation of the membrane integrity and intracellular leakage, as observed on the SEM [18]. Another study demonstrated the wrapping effect of GO on *S. aureus* but not *P. aeruginosa* on AFM [14]. It was speculated to be attributed to the interaction with the thick peptidoglycan of *S. aureus* [50]. Once again, another study also showed the stronger entrapping ability of GO on *S. aureus* than *E. coli*. It was suggested that the smaller and spherical *S. aureus* was easier to entrap [17]. Moreover, another study proposed the bacteria-entrapping effect as the antibacterial mechanism against various multidrug-resistant bacteria. It was suggested to be due to the similar or slightly larger size of GO, which allowed bacteria entrapment [16]. One other study also mentioned that GO nanosheets larger than the bacteria have enhanced entrapping activity. AFM observation showed no change in the morphology, which confirmed the bacteria entrapment [34]. Thus, it can be seen that the size and shape of bacteria play a role in the bacteria-entrapping mechanism. 

In addition, another study also reported on the better entrapping effect of GO on *S. aureus* compared to *P. aeruginosa* [51]. On the other hand, one study demonstrated the bacteria entrapping effect of GO on *P. syringae* and *X. campestris pv. Undulosa*. It was suggested that GO interwind the bacteria blocking the transport across the membrane, leading to cell death [37]. FESEM imaging from another study was also evident in the entrapment of *E. coli* GO [42]. It was found that the entrapment of Gram-negative bacteria occurs at higher GO concentrations: 500 µg/mL [37] and 100 µg/mL [42]. Interestingly, another recent study reported differential antibacterial action GO on Gram-negative and Gram-positive bacteria. This study claimed that the entrapping activity was more effective on Gram-positive *S. aureus* and *E. faecalis* [52]. The results were also suggested to be due to interactions with the peptidoglycan layer. The presence of amino acids, teichoic acids and lipoids on the peptidoglycan facilitates its interaction with GO [53]. Furthermore, it was also mentioned that the entrapping mechanism is more efficient on *S. aureus* than *E. coli*. This was said to be due to presence of certain cell surface molecules, which promotes adsorption onto the GO nanosheet. High surface energy also promotes bacteria entrapment by GO [34]. Therefore, it may be perceived that the entrapping effect of GO is more effective on Gram-positive bacteria.

### 3.3. Oxidative Stress

Oxidative stress caused by GO can be via the production of ROS of the depletion of antioxidants present in the bacterial cell [54]. One recent study has reported that the size of GO does not affect the oxidative stress activity ability on *S. mutans* [16]. Interestingly, a previous study demonstrated that smaller GO nanosheets coated on a nitrocellulose filter surface are better at inducing oxidative stress on *E. coli* [38]. This was also observed in another study [55]. This was speculated to be due to the higher defect densities on the carbon structure, which allowed more oxygen adsorption onto the GO nanosheets. [56]. This was indicated by the higher D band-to-G band ratio of Raman spectroscopy. Interesting, this ratio has a strong correlation with glutathione oxidation (R^2^ = 0.96). It was also mentioned that the oxidation capacity of GO nanosheets does not associate with the amount of oxygen-containing functional groups [38]. One other study also mentioned that the adsorption of oxygen on the defect sites leads to the production of ROS, eventually oxidising glutathione [57]. In addition to this, another study also reported the presence of defects leading to the formation of hydroxyl radicals. Defects were observed by the increase in the D band in the Raman analysis. It was said that the hydroxyl radicals will attack the carbonyl groups in the peptide linkages on the bacterial membrane [22]. It was mentioned that a tighter bacteria entrapping effect and presence of defects improves the interaction between the GO and bacteria, leading to a better charge transfer, increasing oxidative stress [18]. 

Furthermore, another study also suggested the oxidative stress-inducing activity as an antibacterial mechanism for immobilised flat GO on the PET substrate. The immobilised GO renders it unable to penetrate or entrap the *E. coli*. Thus, this study suggested that the oxidative stress was caused by electron transfer via the direct contact of the bacterial with the GO surface [58]. Moreover, another study demonstrated that the availability of the GO basal plane is essential for its antibacterial activity [59]. Nonetheless, a simulation study reported that further oxidation in the oxygen-containing functional groups could improve the oxidation reaction pathways [31]. Moreover, another study reported that GO coated on a metal surface is able to induce oxidative stress via ROS production against *E. coli*. This was said to be attributed to the electron transfer from the bacteria membrane to the oxygen functional groups, generating ROS [36]. On the other hand, it was reported in a study that physical interactions do not play a major role in the antibacterial activity of surface-coated GO. AFM has shown repulsive forces between GO and *E. coli*. In addition, the bridging of lipopolysaccharides on the cell surface also creates a protective barrier. In this study, GO demonstrated its antibacterial activity by the oxidation of glutathione [48]. Therefore, it can be seen that, generally, GO coated on surfaces induces oxidative stress as its main antibacterial mechanism. In contrast, several studies have also shown that ROS produced in bacterial cells exposed to GO is higher than other graphene derivatives. This was suggested to be due to the stability of GO in suspensions [28,60,61]. It was also reported that GO could easily react with water to form hydroxyl radicals, leading to a higher production of ROS [22]. In addition to this, it was also observed that oxidative stress is more effectively induced in Gram-negative bacteria. A few studies have claimed that it was due to the difference in structures of the cell membranes. For example, a study mentioned that Gram-positive bacteria are more susceptible to GO due to the lack of an outer membrane [22]. Several other studies claimed that GO is more effective on Gram-negative bacteria due to the thinner peptidoglycan layer [15,17,20]. Interestingly, another study reported that the peptidoglycan layer does not affect the antibacterial activity of GO [24]. Nevertheless, the antibacterial activity of GO does not depend on the structure of the cell membrane alone. There are other factors that contribute to the antibacterial effect, such as enzymatic activity [62].

## 4. Photoactivation of GO

The antibacterial activity of GO has also been reported to be induced by photoactivation. For example, one study demonstrated that GO reduced to rGO via light induction produces ROS via the singlet oxygen–superoxide anion radical pathway, which was shown to have antibacterial activity against *Enterobacter* sp. [63]. Another study mentioned that *E. coli* was able to kill itself as it reduces GO into rGO in anaerobic conditions [64]. In addition, reduced GO/ZnO nanowires were also reported to have a photo-induced antibacterial effect against *E. coli*. In this study, XPD data showed that GO underwent a photocatalytic reduction due to the charge transfer between GO and ZnO, although the material coated onto ZnO was just GO. Interestingly, the photoinactivation of *E. coli* by reduced GO/ZnO nanowires, which was 99.5%, was signficantly higher than ZnO alone, which was 58% [65]. These studies showed that the reduction of GO can be considered an intermediate pathway in its antibacterial mechanism. It was also noticed that these studies gave GO treatment at the initial stage. This could be due to the lack of dispersibility of rGO in water due to the reduction of hydrophilic functional groups.

## 5. Physicochemical Properties of GO Influence Its Antibacterial Effects

Mechanical defects caused during graphene synthesis can also facilitate its antibacterial activity due to the increase in the number of active sites, which enhances the interaction with bacterial cells [51]. The antibacterial activity of GO also depends on its physicochemical properties. Studies have proven that size, pH, purity, roughness, alignment and type of GO nanosheets also affect the antibacterial potency, as seen in Table 2.

Although smaller-sized GO have been shown to be more effective in the penetration of cell membranes, a chemical interaction-exposed functional group can also lead to oxidative stress. Interestingly, another study mentioned that a higher defect density on the GO nanosheets increases the oxidative potential. In this study, the defects were reported by data from NEXAFS and Raman spectroscopy. The defects were referring to broken bonds and attached oxygen functional groups [42]. Interestingly, a study demonstrated the antibacterial effects of GO coated on a surface, and GO suspension is differentially affected by the size. Coated GO (200 µg/mL) showed an increase in antibacterial activity as the size decreased (0.65 µm^2^–0.01 µm^2^). This was attributed to the more defects exposed leading to higher oxidative potential [38]. These functional groups also increased dispersibility, which enhanced the direct contact with the bacteria and wrapping [45]. In addition, another study has demonstrated that a lower concentration of GO (20 µg/mL), with more functional groups showing a comparable antibacterial effect on *S. mutans* with 40 µg/mL GO, with less functional groups [66]. However, too much oxygen functional groups reduces the electron conductivity. Raman spectroscopy reported that the decrease in size led to a decrease in oxygen functional groups. 

Despite that, the smaller-sized GO possessed higher oxidative potential, as demonstrated by its ability to overcome α-tocopherol, an antioxidant in *E. coli*. This study also reported that bigger-sized GO in suspensions exhibited better wrapping ability. However, the inactivation of *E. coli* by this method was reversible. After the separation of *E. coli* from GO via sonication, colonies were formed on the agar [38]. On the other hand, another study mentioned that the size of GO does not affect the oxidative potential [16]. Interestingly, the size of the bacteria also affects the antibacterial activity of GO [27]. One study has shown that sharp peaks and deep terrains of different GO roughness exhibits the antibacterial activity of different types of bacteria [18]. The topography of GO enables it to interact with the bacteria depending on its size, as wider surfaces allow the bacteria to be swallowed. The physicochemical properties of GO play a pivotal role in the antibacterial mechanism [45]. Therefore, it is essential that the physicochemical properties of GO are the optimum for its antibacterial function. 

## 6. In Vitro Antibacterial Effects of GO

Several studies have also reported on the antibacterial effects of infected cell models. For example, one study showed that GO (10–1000 µg/mL) is able to trap Mycobacterium smegmatis and Mycobacterium tuberculosis in a dose-dependent manner and prevent their entry into J774 murine macrophages. After 4 h, the number of colonies were significantly reduced with 100 µg/mL and 1000 µg/mL GO for M. smegmatis and 1000 µg/mL GO for *M. tuberculosis*. Interestingly, GO did not show antibacterial effects in the infected macrophages and when given prior to infection [49]. This once again suggested that the interaction of GO and the bacteria is extremely crucial. 

Another study demonstrated the effects of GO in P. aeruginosa-infected murine alveolar macrophages. The results showed a dose-dependent effect of GO (0–500 µg/mL) on the viability of the infected macrophages after two hours. The highest macrophage viability was 65% at 250 µg/mL GO and plateaued [11]. It was observed that GO did not exhibit cytotoxic effects on the macrophages in these studies. In another study, GO was also shown to inhibit *S. aureus* in bone marrow mesenchymal stem cells (BMSCs) from murine models. Significant effects were observed at concentrations 50–1000 µg/mL GO at 0, 8 and 24 h. In addition, the cytotoxic effects of GO were also reported on BMSCs at 50–1000 µg/mL. This was indicated by the change in cellular morphology observed in the SEM and TEM [67]. 

## 7. In Vivo Antibacterial Effects of GO

There have been a few studies reporting on the antibacterial activity of GO in vivo. For instance, a study reported the antibacterial activity of 100 µg/mL GO in mice at 0 and 24 h in mice against *S. aureus*. GO also exhibited toxicity in vivo, as indicated by muscular atrophy and necrosis after 7 days [67]. Another study demonstrated that GO (250 µg/mL) is able to inhibit the invasion of P. aeruginosa in mice. After 24 h, the results showed that the bioluminescence of P. aeruginosa decreased by almost two-fold when compared to the untreated mice. Moreover, the results also reported the increase in the survival rate and prolonged survival time of the GO-treated mice. Interestingly, GO was shown to be able to alleviate inflammation, as indicated by the reduced polymorphonuclear neutrophil (PMN) infiltration and tissue damage. Meanwhile, the viability of alveolar macrophages isolated from mice was also increased by 1.4- and 3.1-fold compared to the untreated mice after 24 and 48 h, respectively. Furthermore, the ROS generation, bacterial colony-forming units from each organ and myeloperoxidase activity also decreased after 8 and 24 h [11]. Indeed, not many in vitro and in vivo studies are currently being done on the antibacterial activity of GO. This is because the studies of GO for its antibacterial activities are still at the infancy stage, as it has only been reported since 2010 [68].

## 8. Biocompatibility of GO

One of the major hurdles in fully integrating GO applications in industries, especially biomedicine, is the potential biocompatibility issues that have been reported in the literature. Interestingly, studies have also reported the biocompatibility of GO with human mesenchymal stem cells by inducing proliferation and differentiation into the osteogenic lineage [69,70]. However, there were many reports that suggest GO having significant cytotoxicity in both in vitro and in vivo studies involving various biological models, such as mammalian cells and animal models. In general, graphene family nanomaterials may exhibit varying degrees of a toxicity response in animals and cell models, depending on the route of administration and the penetration of a physiological barrier. The translocation of GO nanomaterials, as well as accumulation in specific tissues/organs and, eventually, clearance of GO nanomaterials through excretion pathways could also affect the toxicity response. 

In one in vitro study conducted by Chang et al. (2011), the toxicity assessment of GO showed no significant observable changes on the morphology, viability, mortality and membrane structure in cells [71]. However, it was noted that GO exposure induces oxidative stress at low concentrations. Furthermore, a study of GO using the MTT assay indicated that, after prolonged treatment, GO was able to induce cytotoxicity and genotoxicity, as well as oxidative stress, which eventually leads to a significant decrease in cell viability, as well as apoptotic cell death [72]. On the other hand, the in vivo toxicity study of GO reported that there was no significant toxicity effect when administered intravenously into mice at a low dosage. However, at a high dosage, chronic toxicity was observed. Interestingly, a histopathology analysis carried out showed that GO accumulation was detected in the lungs, liver and spleen of the mice, which correlates to organs that are most like to take up and sequester foreign materials. Further analysis of the dead mice also showed blockage of the airway by GO accumulation [73].

There is an increase in the number of studies recognizing the need to study the influence of the lateral dimensions of graphene oxide to the potential toxicity effect of GO. Yue et al. (2012) demonstrated that the uptake of GO was independent of the lateral dimensions. GO samples with different lateral dimension, 2 µm and 350 nm, were tested against six different cell lines, of which only two were able to internalise GO (phagocytosis). Although the differences in lateral dimensions were quite drastic, the final uptake of GO was relatively similar, leading researchers to conclude that the cellular uptake of GO is size-independent (lateral dimension), although, in this study, the GO used were between three and four layers thick [74]. Another study by two researchers showed that the toxicity of GO was contrastingly dependent on the size or lateral dimensions of GO. They prepared three different GO suspensions with sizes ranging from 150 to 1000 nm that were exposed to several different cell lines. Their results found that GO with larger sizes in general induce less toxicity effects in cells, while small- and medium-sized ones induced a significantly higher toxicity. This may be due to small- and medium-sized GO flakes readily experienced cellular uptake, which induced a higher toxicity and vice versa [75].

Several studies have also shown that the toxicity effect of GO is influenced by the dosage level or concentration. In one study, Chen et al. (2012) demonstrated that the toxicity of GO is influenced by the administered dosage. They assessed the cytotoxicity of GO using the Cell Counting Kit-8 (CCK-8) assay and employing the human bone marrow neuroblastoma cell line (SK-N-SH) and human epithelial carcinoma cell line (HeLa). The cell lines were exposed to varying concentrations of GO prepared in an egg–water solution ranging from 0, 3.4, 7.6, 12.5, 25 to 50 mg/L. They showed that administered GO with concentrations between 3.4 and 25 mg/L did not stop the growth or proliferation of SK-N-SH, while, at 50 mg/L, the growth was inhibited by 20%. GO, on the other hand, exhibited only minimal cytotoxicity in HeLa cells (cancer cells) proliferation, even at 50 mg/L [76]. In 2014, Liu et al. conducted a study involving zebrafish embryos that were exposed to different concentrations of GO at concentrations ranging from 0, 1, 5, 10, 50, to 100 mg/L suspended in standard water. The researchers exposed these different concentrations of GO to zebrafish embryos at 48 h post-fertilization (hpf). The toxicity effect was demonstrated by observing the heartbeat and hatching rate, as well as the larvae length. When exposed to 100 mg/L of GO at 48 hpf, zebrafish embryos experienced a significant drop in heartbeat, while, at 96 hpf, the heartbeat of zebrafish larvae showed a dose-dependent decrease from 5 to 100 mg/L of GO exposure. The same study also showed that, as the concentration of GO increases, GO coating the surface of the chorion of zebrafish embryo increases as well, which was not surprising. However, the study indicated that GO did not show any significant effect on the hatching rate or larval length of the zebrafish embryos. The researchers noted that this was probably due to the fact that GO did not adhere to the chorion of the embryo tightly due to the structural properties of GO [77]. In 2015, another group of researchers exposed zebrafish embryos to 0.01, 0.1, 1.0, 10, and 100 mg/L of GO suspension in E3 embryo culture medium [78]. They noted that mortality did not increase the noticeably in any of the GO-treated groups when compared to the control group. However, a decrease in heart rate was recorded for all groups at 48 hpf except at 100 mg/L, where the heart rate increased. There was a significant decrease in the heart rate for the groups exposed to 1 mg/L [78]. The inconsistent results between the two studies conducted might be attributed to the differences in the average sizes of the GO nanosheets in both studies. As for the hatching rate, there was a drastic decrease in group exposed to 100 mg/L after 96 hpf. Aside from this, the apoptosis occurrence and reactive oxygen species (ROS) were also observed. The apoptosis level and ROS generation were significantly increased over the whole body in a concentration-dependent manner, with a marked increased around the eye, heart and tail regions.

The time-dependent toxicity effect of GO was also studied to observe the relationship between exposure time and cytotoxicity. Aside from looking at the effect of different concentrations of GO, Liu et al. (2014) also studied the time-dependent effect of GO toxicity. The same range of the concentration (0, 1, 5, 10, 50, and 100 mg/L) was administered externally to zebrafish larvae at 96 hpf, and the heartbeat was measured. While, at 48 hpf, the heartbeat of zebrafish embryos only decreases significantly at 100 mg/L, the zebrafish larvae at 96 hpf exhibited a decrease in the heart rate from 5 to 100 mg/L in a dose-dependent manner. However, as mentioned previously, this did not have any noticeable effect on the hatching rate, as well as larval length. Another in vivo study was also conducted, which involved 2-month-old healthy adult zebrafish. The zebrafish, after being acclimatized for 1 week prior, were exposed to 0, 1, 5, and 10 mg/L GO for a 14-day period. The survival rate of the zebrafish was found to be not significantly affected [77].

There is adequate evidence that GO has been shown to possess a significant level of toxic potential when studied in in vitro and in vivo systems. However, the functionalization of GO through surface coating has shown positive correlations between GO surface functionalization and reduced the toxicity effect/improved the toxicity profile. One of the many potential surface coatings that were studied was dextran (DEX), a type of glucose polymer, bovine serum albumin (BSA) and polyethylene glycol (PEG). A research group, Kim et al., coated GO with DEX and reported that GO–DEX was highly biocompatible towards the HeLa cell line, with the cell viability remaining at 80%, even at concentrations of up to 450 µg/mL [79]. In addition, in vitro studies conducted using BSA as a surface coating on GO have shown that it could improve GO biocompatibility, with only a slight inhibition towards cell proliferation up 50 µg/mL. This was attributed to protein adsorption to GO, which weakened the GO interactions towards the cells [80,81]. In addition to that, another in vitro study also demonstrated that the GO–PEG conjugate was highly biocompatible towards A549 cells, with no cytotoxicity response observed at concentrations of up to 100 µg/mL after a 48-h incubation period. GO has also been shown to have improved toxicity upon functionalization with gallic acid, a phenolic compound. A higher LC_50_ value of more than 500 μg/mL was recorded for gallic acid-loaded GO (GAGO) following 72-h exposure in zebrafish embryos [82] without comprosing the antibacterial effects of GO towards MDR bacteria [83]. A previous study from our research group has also demonstrated a significant improvement in the biocompatibility of GO upon functionalization with a nonionic surfactant, Pluronic F127, that suggests a promising approach to mitigate the toxicological response of GO and solve the biocompatibility issue of GO [84]. 

## 9. Conclusions and Future Directions

It is very evident that GO is able to impose potent antibacterial activity through various potential mechanisms of action. However, the antibacterial activity and development of GO in clinical settings have been significantly influenced by its physicochemical properties and its ambiguous toxicity. Therefore, further research should also take into consideration the method of preparation, which affects the physicochemical properties of GO that could directly impact its antibacterial activity. Studies should also try to bridge the gap between studies instead of adding to the pool of similar studies with incomparable parameters. In addition to that, the prospective utilisation of GO requires an even more thorough assessment to ensure its biocompatibility, in which the concentration of GO that demonstrates the optimum antibacterial activity must have the minimum cytotoxicity. Moreover, studies should also be conducted on the antibacterial activity of varying degrees of oxidation of GO against different MDR bacteria, due to the differences in cell wall compositions. With that, GO could potentially be developed into an antibacterial therapeutic in the future.

## Figures and Tables

**Table 1:** Antibacterial activity of GO reported as time and concentration-dependent.

Findings	Concentration	Time	Reference
Results showed concentration-dependent decrease in the survival rate of *K. pneumoniae*, *E. coli* and *P. aeruginosa*. Antibacterial effect was most effective on *K. pneumoniae*. This was evident with bioluminescence indicating live bacteria.	0–500 µg/mL	2 h	[11]
The MIC for *E. coli*, *K. pneumoniae*, *P. mirabilis* and *S. aureus* is 0.065 µg/mL. The MIC for *P. aeruginosa* and *S. marcescens* is 0.032 µg/µL. The MBC of *P. aeruginosa* and *S. marcescen*s is 0.065 µg/mL. The MBC for *E. coli*, *K. pneumoniae*, *P. mirabilis* and *S. aureus* is 0.12 µg/mL.	0.004–1 µg/mL	24 h	[12]
Results showed concentration-dependent increase in the zone of inhibition for *E. coli* and *S. aureus*. Zone of inhibition was bigger on *S. aureus*.	250–1000 µg/mL	24 h	[13]
Results showed thesignificant growth inhibition of *S. aureus* at 2 and 24 h and for *P. aeruginosa* at 2 h.	50 mg/L	2 and 24 h	[14]
MIC for *E. coli* and *E. faecalis* was 1 µg/mL and 4 µg/mL, respectively.	-	24 h	[15]
Results showed that the percentage loss in viability increases as the concentration and time increases.	12–50 µg/mL	30–180 min	[16]
Results showed concentration- and time-dependent decreased viability of bacteria. The significant reduction of viability of *S. aureus* was 12 h, while, for *E. coli*, it was 168 h.	0–40 mg/mL	-	[17]
After 15 min, there was 99% loss in viability of *Mycobacterium smegmatis*, *E. coli* and *S. aureus*.	1 mg/mL	15 min in the dark	[18]
Results showed a decrease in the recovery of *E. coli*.	1 mg/mL	2 and 4 h	[19]
The survival rate of *E. coli* is 4% at 100 µg/mL. Results also showed that the average growth delay of exponential cells varies around 4 to 6 h. *E. coli* and *P. aeruginosa* also showed alow survival rate. On the other hand, a bacteriostatic effect was observed on *S. aureus*, as indicated by the high survival rate.	0–100 µg/mL	30 min	[20]
Results showed time-dependent decrease in the viability of *E. coli*.	0.1 g/L	0–5 h	[21]
The MIC and EC_50_ on *E. coli* are 100 µg/mL and 38 µg/mL, respectively. The MIC and EC_50_ on *S. iniae* are 125 µg/mL and 29 µg/mL, respectively.	25–150 µg/mL	3 h	[22]
Results showed a time-dependent loss in viability of *P. aeruginosa*, and the percentage loss at 4 h is 87%. There was a concentration-dependent loss in viability that reached a complete loss at 175 µg/mL. Bacterial growth was shown to increase and decrease after 3 h at 75 µg/mL. There was a 92% growth inhibition at after 15 h.	0–200 µg/mL	2 h	[23]
Results showed the concentration- and time-dependent decreases in viability of *E. coli*.	0–80 µg/mL Time study: 40 µg/mL	2 h	[24]

**Table 2:** Antibacterial activity exhibited by GO depends on its physicochemical properties.

Findings	Nature of GO	Concentration, Time	Reference
The size of GO flakes affects the antibacterial activity on *S. mutans*. It was shown that GO-1 demonstrated better cutting activity, while GO-2–4 were better at entrapping bacteria as the size increases. GO-2 is most effective with the combination of cutting and entrapping activity.	Size: GO-1 (2 µm), 2 (4 µm), 3 (6 µm), 4 (8 µm)	25 µg/mL, 10 s	[16]
GO with roughness of 505 nm decreased 20% viability of *E. coli* and *S. aureus*, while 845 nm GO decreased 30% viability of *M. smegmatis*. SEM showed wrapping and leakage of intracellular substances. The cell membranes were completely decomposed. There was a significant decrease in cell volume. If the surface roughness of GO nanosheets was consistent with the diameter of bacteria, this increases contact area and bacterial adhesion. As the GO nanosheet looks like a mountain range, the peaks improve charge transfer causing destruction of the bacterial cell membrane leading to intracellular leakage. Deep terrains trap bacteria of matching diameter, enhancing the strong interaction with the bacterial cells and promoting direct oxidation of cellular components. Fluorescent staining also indicated leakage of DNA due to a compromised membrane.	Roughness: 465, 505, 845, 1179 nm	1 mg/mL, 15 min in the dark at room temperature	[18]
Results showed that increase in washes increased the recovery of *E. coli.* Interestingly, highly purified GO does not affect growth curve of *E. coli* and *S. aureus* and rather showed concentration-dependent increase in the growth rate (10–250 µg/mL). Size of highly purified GO does not affect growth rate (50 µg/mL) and morphology (100 µg/mL) of *E. coli*.Results suggested that increase in pH decrease the bactericidal effects. It may also be due to chemical contaminants present in the GO preparations as a consequence of the generation of the GO. Moreover, highly purified GO had no effects on growth or inhibition of bacteria.	GO washes: 2 (pH 3.5), 4 (pH 4), 6 (pH 5), 8 (pH 5.5)	1 mg/mL, 2 and 4 h at 30 °C.	[19]
Survival rate of exponential *E. coli* is 4%. Average growth delay of exponential cells is longer than those in stationary and decline phases for *E. coli*, *S. aureus* and *P. aeruginosa*. In conclusion, bacteria is more susceptible to GO at the exponential phase. It was mentioned that as bacteria gradually matures through the growth phases, they generate phenotypically different subpopulations.	Cells in exponential, stationary, decline phases.	100 µg/mL, 30 min	[20]
GO foam demonstrated more efficient antibacterial activity on *E. coli* than GO precipitate.	GO foam and GO precipitate	0.1 g/L, 0–5 h	[21]
Increase size GO, decreased the viability of *E. coli*. Results showed that larger GO showed efficient antibacterial effects with lower concentrations of GO (10 µg/mL) and less treatment time (1 h).	Sizes of GO (µm^2^) GO-0: 0.753 GO-10: 0.127 GO-30: 0.065 GO-50: 035 GO-120: 0.013 GO-240: 0.010	40 µg/mL, 2 h	[24]
Results showed enhanced antibacterial activity on *E. coli* through the controlled alignment of GO nanosheets leading to cell membrane disruption and oxidative stress caused by electron transfer. Vertically aligned GO was the most effective.	Random, vertical, planar alignment	200 µg/mL, 3 h	[29]

## Data Availability

Not applicable.
